# Hypomorphic variants of cationic amino acid transporter 3 in males with autism spectrum disorders

**DOI:** 10.1007/s00726-015-2057-3

**Published:** 2015-07-28

**Authors:** Caroline Nava, Johanna Rupp, Jean-Paul Boissel, Cyril Mignot, Agnès Rastetter, Claire Amiet, Aurélia Jacquette, Céline Dupuits, Delphine Bouteiller, Boris Keren, Merle Ruberg, Anne Faudet, Diane Doummar, Anne Philippe, Didier Périsse, Claudine Laurent, Nicolas Lebrun, Vincent Guillemot, Jamel Chelly, David Cohen, Delphine Héron, Alexis Brice, Ellen I. Closs, Christel Depienne

**Affiliations:** Sorbonne Universités, UPMC Univ Paris 06, UMR S 1127, ICM, 75013 Paris, France; INSERM, U 1127, 75013 Paris, France; CNRS, UMR 7225, 75013 Paris, France; Institut du cerveau et de la moelle épinière (ICM), 75013 Paris, France; Département de Génétique et de Cytogénétique, Hôpital de la Pitié-Salpêtrière, AP-HP, 75013 Paris, France; Department of Pharmacology, University Medical Center of the Johannes Gutenberg University, Mainz, Germany; Centre de Référence “déficiences intellectuelles de causes rares”, Paris, France; Groupe de Recherche Clinique (GRC) “déficience intellectuelle et autisme” UPMC, Paris, France; Service de neuropédiatrie, Hôpital Trousseau, AP-HP, Paris, France; Service de psychiatrie de l’enfant et de l’adolescent, Hôpital Pitié-Salpêtrière, AP-HP, 75013 Paris, France; Centre Diagnostic Autisme de l’Hôpital Pitié-Salpêtrière, 75013 Paris, France; Institut Cochin, Inserm U567, UMR 8104, Université René Descartes, Paris 5, France; Bioinformatics and Biostatistics Core Facility (iCONICS), Institut du cerveau et de la moelle épinière (ICM), Paris, France; Institut des Systèmes Intelligents et Robotiques, CNRS UMR 7222, UPMC-Paris-6, Paris, France

**Keywords:** Cationic amino acid transporter, Autism spectrum disorders, Exome sequencing, Chromosome X, Oligogenism

## Abstract

Cationic amino acid transporters (CATs) mediate the entry of L-type cationic amino acids (arginine, ornithine and lysine) into the cells including neurons. CAT-3, encoded by the *SLC7A3* gene on chromosome X, is one of the three CATs present in the human genome, with selective expression in brain. *SLC7A3* is highly intolerant to variation in humans, as attested by the low frequency of deleterious variants in available databases, but the impact on variants in this gene in humans remains undefined. In this study, we identified a missense variant in *SLC7A3*, encoding the CAT-3 cationic amino acid transporter, on chromosome X by exome sequencing in two brothers with autism spectrum disorder (ASD). We then sequenced the *SLC7A3* coding sequence in 148 male patients with ASD and identified three additional rare missense variants in unrelated patients. Functional analyses of the mutant transporters showed that two of the four identified variants cause severe or moderate loss of CAT-3 function due to altered protein stability or abnormal trafficking to the plasma membrane. The patient with the most deleterious *SLC7A3* variant had high-functioning autism and epilepsy, and also carries a de novo 16p11.2 duplication possibly contributing to his phenotype. This study shows that rare hypomorphic variants of *SLC7A3* exist in male individuals and suggest that *SLC7A3* variants possibly contribute to the etiology of ASD in male subjects in association with other genetic factors.

**Electronic supplementary material:**

The online version of this article (doi:10.1007/s00726-015-2057-3) contains supplementary material, which is available to authorized users.

## Introduction

The main function of cationic amino acid transporters (CAT) is to mediate the entry of l-type cationic amino acids (i.e., l-arginine, l-ornithine and l-lysine) into many different cell types including neurons (Closs et al. 2006; Jager et al. 2013). Their function is crucial since lysine and arginine, under certain conditions, are essential amino acids that are derived exclusively from the degradation of ingested nutrients. The CAT family comprises three different genes: *SLC7A1*/CAT-1 on chromosome 13, *SLC7A2*/CAT-2 on chromosome 8, and *SLC7A3*/CAT-3 on chromosome X. All three transporters have different and complementary tissue localizations, making each of them necessary for life and normal health. CAT-3 is selectively expressed in brain in rodents (Hosokawa et al. 1997; Ito and Groudine 1997). In neurons, CAT-3 responds to NMDA receptor activation and regulates the mammalian target of rapamycin (mTOR) signaling pathway, which has a central role in neuronal development and plasticity, through arginine availability (Huang et al. 2007).

Autism spectrum disorders (ASD) are neurodevelopmental disorders characterized by impaired social interactions and communication, restricted interests and repetitive or stereotyped behaviors (Lai et al. 2014). Intellectual disability (ID) is a frequent comorbidity of ASD, present in more than half of ASD subjects (Srivastava and Schwartz 2014; Tuchman and Rapin 2002; Amiet et al. 2008). ASD are highly genetically determined, but the genetic factors involved in these disorders are extremely heterogeneous and have proven difficult to identify (Betancur 2011; Huguet et al. 2013; Jeste and Geschwind 2014), and, in spite of the acceleration of gene identification due to technological advances, a genetic cause is still found in a minority of ASD cases. De novo or inherited copy number variants (CNV), strongly associated with autism and probably conferring high susceptibility to ASD, have been identified in 2–10 % of patients (Girirajan et al. 2013; Sanders et al. 2011; Glessner et al. 2009; Bucan et al. 2009; Pinto et al. 2010; Huguet et al. 2013). Additional copies of the 15q11–q13 region or an abnormal number of copies in the 16p11.2 region are examples of recurrent CNVs found in ASD (Sanders et al. 2011; Depienne et al. 2009; Weiss et al. 2008; Kumar et al. 2008; Nava et al. 2014b; Levy et al. 2011). More recently, exome sequencing of parent–offspring trios has shown that de novo point mutations contribute to ASD in 10–30 % of sporadic patients (Murdoch and State 2013; Krumm et al. 2014; O’Roak et al. 2011, 2012; Sanders et al. 2012; Neale et al. 2012; Iossifov et al. 2012, 2014). These studies predicted that ASD could result from genetic abnormalities in several hundreds of different genes, many of which are, nonetheless, interconnected or part of common functional pathways (Neale et al. 2012; O’Roak et al. 2012; Sanders et al. 2012; Iossifov et al. 2012; Gilman et al. 2011). Examples of pathways repeatedly involved in ASD include: synaptic function, illustrated by mutations in SHANK1-3 scaffolding proteins, neuroligins, neurexins, contactins and contactin-associated proteins encoding genes; the mTOR pathway, illustrated by mutations in *TSC1/TSC2* or *PTEN* that cause syndromic forms of ASD; chromatin remodeling; and Wnt signaling (Krumm et al. 2014; Jeste and Geschwind 2014; Huguet et al. 2013). An excess of males (4 affected males for one affected female) is typically observed in ASD (Schaafsma and Pfaff 2014; Werling and Geschwind 2013), suggesting that genes located on sex chromosomes contribute to the etiology of the disorders, or that the penetrance of autistic traits depends on sex determinants (Werling and Geschwind 2013).

In this study, we used exome sequencing to identify genetic factors contributing to ASD in a family comprising two affected brothers. The identification of a missense variant in *SLC7A3* on chromosome X, shared by the two brothers, prompted us to investigate the consequences and phenotypic contribution of variants in this gene in male individuals.

## Materials and methods

### Subjects

**Fig. 1:**
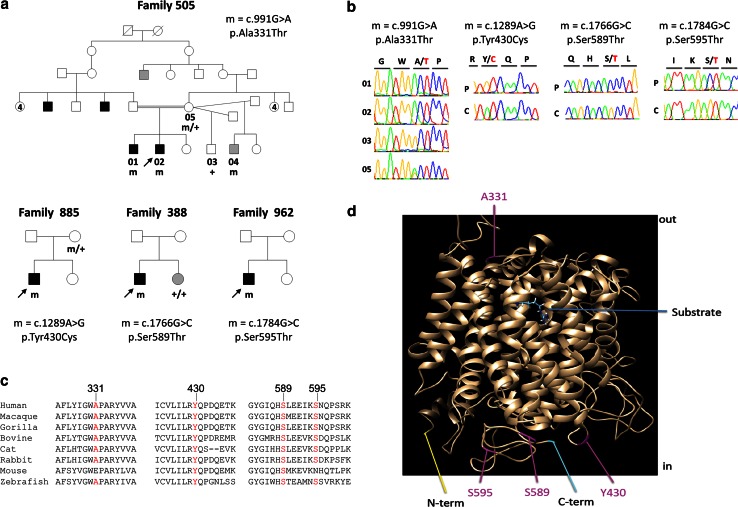
Identification of missense variants in *SLC7A3* in four patients with ASD. **a** Pedigree of the families and segregation of *SLC7A3* variants; *m* and *m/+* denotes male or female individuals carrying one variant in the hemizygous or heterozygous state, respectively. *Arrow* the index case. *Black* *symbols* individuals diagnosed with ASD; *gray symbols* patients with undetermined intellectual disability or learning difficulty phenotypes (see details in Supplementary data). **b** Sequence electropherograms showing the *SLC7A3* variants in the hemizygous state in the affected individuals of Families 505 (01, 02), 885, 388, 962 (P), in the heterozygous state in the mother of Family 505 (05) and their absence in an unaffected brother in Family 505 (03) and controls (C). **c** Alignment of the regions flanking the *SLC7A3*/CAT-3 missense variant in orthologous proteins showing the conservation of the altered amino acids. **d** Schematic model of the CAT-3 protein showing the putative location of the amino acid residues altered by the variants

Exome sequencing was performed in Family 505, originating from Morocco, and comprising two affected brothers born of consanguineous parents (Fig. 1a). The proband (01) had a normal motor development but presented with language delay. He was diagnosed with autistic spectrum disorder associated with moderate intellectual disability. He had obsessive–compulsive behaviors, phobias and sleeping difficulties. His affected brother (02) had a clinical history similar to that of his older brother but he had more severe intellectual disability and never acquired language (Supplementary material). A younger half-brother had a language delay at the age of 3 years, and 2 maternal cousins had ID with unspecified behavioral disturbances. DNA was unavailable from the father and the cousins.

Sequencing of *SLC7A3* was then performed in 148 male subjects with ASDs recruited in the “Centre de Référence Déficiences Intellectuelles de Causes Rares” and the “Centre Diagnostic Autisme” (Pitié-Salpêtrière Hospital, Paris, France) (Nava et al. 2014b). Index cases were assessed with the Autism Diagnostic Interview-Revised (ADI-R) and had ASD based on DSM IV-TR criteria: 120 index cases (81 %) had autism with ID and 28 (19 %) had Asperger syndrome or high-functioning autism; 82 % (122/148) of ASD patients were sporadic cases.

The study was approved by the local Institutional Review Board (Comité de Protection des Personnes, Hôpital Pitié-Salpêtrière, Paris, France). Informed written consent was obtained from each subject or his parents or legal representatives before blood sampling. Genomic DNA of patients and relatives was extracted from blood cells using standard phenol–chloroform procedures. Cerebrospinal fluid (CSF) sampling in subject 02 of family 505 was performed in a diagnostic context.

### SNP array analysis

The affected brothers and their healthy sister were genotyped using cytoSNP-12 microarrays (Illumina, San Diego, CA). Automated Illumina microarray experiments were performed as previously described (Nava et al. 2014b). Image acquisition was performed using an iScan System (Illumina). Image analysis and automated CNV calling was performed using GenomeStudio v2011.1 and CNVPartition v3.1.6 with the default confidence threshold of 35. Loss of heterozygosity (LOH) regions with a size >2 Mb were determined using CNVPartition v3.1.6.

### Exome sequencing

The exome of the two affected brothers in family 505 and their unaffected mother was sequenced by Integragen SA (Evry), as previously described (Nava et al. 2014a). Rare coding variants or variants predicted to alter consensus splice sites with a read depth ≥10 shared by the two affected brothers were listed using the ERIS interface (Integragen). Rare variants were defined by a minor allele frequency (MAF) ≤1 % in Hapmap, 1000 Genomes, Exome variant server, and in an in-house Integragen exome database. Further analysis of exome data focused on the search for homozygous mutations located in identical-by-descent regions or hemizygous variants on chromosome X. Possibly deleterious variants were defined as indels introducing frameshifts or in-frame insertions or deletions, nonsense or splice-site mutations, mutations altering start or termination codons, or nonsynonymous variants predicted to be possibly deleterious by at least one of three prediction algorithms (see bioinformatics analyses).

### Sanger sequencing

Specific primer pairs were designed to confirm the variants detected by exome sequencing in *SLC7A3, CCDC120, ARAF, FAM123B* and *SLC9A6* on chromosome X, and *SCN2A, MAS1L, FOXP2, ROBO4, NOS1, PARP4, CACNAIH, ZSCAN10, TRAP1* and *TMPRSS9* on autosomal chromosomes and to study their segregation in relatives. The exons and intron–exon junctions of *SLC7A3* (NM_001048164.2) were amplified and analyzed using 11 primer pairs (Table S1). Forward and reverse sequence reactions were performed with the Big Dye Terminator Cycle Sequencing Ready Reaction Kit (Applied Biosystems, Foster City, California). G50-purified sequence products were run on an ABI 3730 automated sequencer (Applied Biosystems); the data were analyzed with Seqscape v2.6 software (Applied Biosystems).

### Bioinformatic and statistical analyses

Missense variants were assessed in silico for possible pathogenicity using Alamut 2.3 (Biointeractive Software, France), PolyPhen-2 (http://genetics.bwh.harvard.edu/pph2), SIFT (http://sift.bii.a-star.edu.sg), and Mutation Taster (www.mutationtaster.org). A three-dimensional model of the first predicted 10 TMDs of hCAT-3 was generated as previously described for hCAT-2A (Beyer et al. 2013). Comparison of the number of *SLC7A3* variants in male ASD patients versus males of the ESP population (*n* = 2443, Exome variant server, http://evs.gs.washington.edu/EVS/) or male control subjects included in the IPDGC study (*n* = 338) was performed with the Fisher’s Exact Test. The probability to identify at least n variants in *SLC7A3* in the tested patient population was calculated based on the frequency of *SLC7A3* rare variants in the ESP and IPDGC populations using a binomial distribution.

### Immunofluorescence staining and isolation of plasma membrane proteins

Missense variants identified in autistic patients were introduced into plasmids expressing the human CAT-3 cDNA fused to the Green Fluorescent protein (GFP). Cos7 cells were transiently co-transfected with 5 µg of WT or mutant CAT-3 expression plasmids using a neon electroporation system (Invitrogen). Cells were fixed with 4 % paraformaldehyde (PFA) 24 h after transfection, permeabilized with 0.1 % Triton X-100, and incubated with anti-calreticulin (ER marker, Abcam, ab2907, 1:1000) for at least 2 h at room temperature. The signal was revealed by incubation with a Cy3-coupled sheep anti-mouse IgG antibody (Sigma, 1:1000) for 1 h at room temperature. Nuclei were stained with Hoechst (1:1000). Fluorescent images were acquired with a confocal system (Leica SP2 AOBS AOTF).

Proteins present at the plasma membrane of Cos7 transfected cells were isolated following surface biotinylation of living Cos7 cells with the Cell Surface Protein Isolation Kit (Pierce), following the manufacturer’s recommendations. Proteins were resolved by SDS-PAGE on 4–12 % gradient gels (Invitrogen) and electrotransferred onto nitrocellulose membranes. CAT-3 was probed with an anti-GFP antibody (monoclonal mouse anti-GFP antibody, #11814460001, Roche, 1:4000), and the signal was revealed by enhanced chemiluminescence (Pierce). The membranes were subsequently probed with an anti-Tom20 (BD Biosciences 612278, 1:1000) antibody to confirm plasma membrane enrichment, and with an anti-Flotillin-1 (BD Biosciences 610820, 1:1000) antibody for normalization. The ImageJ program (http://rsb.info.nih.gov/ij/) was used for signal quantification. Independent measures from at least 3 different experiments were analyzed with the Mann–Whitney test.

### Transporter expression and transport studies in *Xenopus laevis* oocytes

cRNA was prepared by in vitro transcription from the SP6 promoter of CAT-3-GFP-pSP64T (mMessage mMachine in vitro transcription kit, Ambion Inc, Austin, TX, USA) (Vekony et al. 2001). Seventeen nanograms of human CAT-3-GFP cRNA were injected into each *X. laevis* oocyte (Dumont stages V–VI). Noninjected oocytes were used as controls. Arginine uptake was determined 3 days after injection of cRNA as previously described (Closs et al. 1997). Briefly, oocytes were washed in uptake solution (100 mM NaCl, 2 mM KCl, 1 mM MgCl_2_, 1 mM CaCl_2_, 5 mM HEPES, 5 mM Tris, pH 7.5) containing 1 mM unlabeled arginine then transferred to the same solution supplemented with 0.37 MBq/ml L-[2,3,4-^3^H]arginine monohydrochloride (MP), 1.59TBq/mmol. After incubation for 15 min at 20 °C, the oocytes were washed four times in ice-cold uptake solution and solubilized individually in 2 % sodium dodecyl sulfate (SDS). The incorporated radioactivity was quantified in a liquid scintillation counter (Tri-Carb 2810 TR, Perkin Elmer).

### Oocyte lysates and biotinylation of cell surface proteins

All steps were performed at 4 °C, as previously described (Beyer et al. 2013). Briefly, ten oocytes were each incubated for 30 min with membrane impermeable EZ-Link™Sulfo-NHS-SS-Biotin (Sulfosuccinimidyl-2-(biotinoamido)ethyl-1,3-dithiopropionate, Thermo Fisher Scientific Inc., Rockford; 1 mg/ml in PBS_mod_/CM). The biotinylation reaction was stopped by incubating the oocytes in PBS_mod_ containing 50 mM NH_4_Cl for 10 min. After lysis in 200 μl radioimmune precipitation assay buffer (RIPA: 1 % deoxycholate, 1 % Triton X-100, 0.1 % SDS, 150 mM NaCl, 2 mM MgCl_2_, 10 mM Tris–HCl pH 7.2) containing protease inhibitors (Complete Mini EDTA-free protease inhibitor tablets, Roche, Basel), an aliquot of each whole oocyte lysate was mixed directly with an equal volume of 2 × sample buffer (125 mM Tris base, 20 % glycerol (v/v), 5 % SDS, 0.001 % bromphenol blue (m/v), 8 M urea, 2 % mercaptoethanol) and incubated for 10 min at 37 °C. The remaining lysate was incubated overnight with avidin-coated Sepharose beads (NeutrAvidin^®^ UltraLink^®^ Resin, Thermo Fisher Scientific Inc., Rockford) to recover the biotinylated surface proteins. The beads were then washed three times with RIPA containing protease inhibitors (PMSF 200 µM). Biotinylated proteins were released from the beads by incubation in 2× sample buffer for 10 min at 37 °C. Oocytes lysates were then separated by 7.5 or 12.5 % SDS-PAGE and tank-blotted onto nitrocellulose membranes, as previously described (Beyer et al. 2013). CAT-3 proteins on the blots were stained by incubation with rabbit polyclonal GFP antibody (Clontech Living colors #632460, 1:3000) overnight at 4 °C followed by goat anti-rabbit IgG, H&L chain-specific peroxidase conjugate (Calbiochem #401393, 1:15,000) for 1 h at room temperature. The blots were then incubated for 1 min with the chemiluminescence reagent (Western Lightning^®^ ECL-Plus, Perkin Elmer, USA) and exposed to chemiluminescence films (Hyperfilm ECL, GE Healthcare Life Sciences, UK). Signal intensity was quantified using Chemidoc^®^ XRS with Quantity One software (BioRad, Berkeley, USA). For standardization, membranes were stained with a mouse monoclonal anti-ß-tubulin antibody (T 4026, Sigma-Aldrich, Deisenhofen, 1:5000) and a rabbit anti-mouse IgG peroxidase conjugate (A 9044, Sigma-Aldrich, Deisenhofen, 1:5000).

## Results

Family 505 comprises two brothers with ASD born from North African consanguineous parents. To identify variants contributing to ASD, we sequenced the exome of the brothers and their healthy mother (Fig. 1a). The affected brothers and their healthy sister were genotyped, in parallel, using Illumina SNP arrays. No pathogenic CNV were detected by this analysis in the affected sibs. Two LOH regions shared by the affected brothers and absent from their sister, a 2.3 Mb region on chromosome 8 and a 7 Mb region on chromosome 15, containing 28 and 39 genes, were found (Fig. S1 and Table S2). Exome sequencing detected 351 rare variants shared by the affected brothers that altered the coding sequence or consensus splice sites in 329 genes (Table S3). None of the variants was located in LOH regions.

Since two maternal male cousins were reported to have unspecified ID and behavioral disturbances, we decided to focus our study on X-chromosomal variants. Three nonsynonymous variants predicted to be possibly deleterious by at least one prediction tool were located on chromosome X (c.624G > C/p.Gln208His in *ARAF*, c.991G > A/p.Ala331Thr in *SLC7A3,* c.1477G > A/p.Ala493Thr in *CCDC120*). Analyses of North African control subjects showed that the frequency of the variants in *ARAF* and *CCDC120* was higher than reported in databases in other populations, making their involvement in the phenotype of the brothers unlikely; the c.991G > A/p.Ala331Thr variant in *SLC7A3* was not found, however, in 630 controls including 440 North African subjects (Table S4).

We then screened 148 unrelated males with ASD for mutations in exons of *SLC7A3*. We identified three rare hemizygous variants that altered conserved amino acids in three patients (Fig. 1): c.1289A > G/p.Tyr430Cys was identified in a 10-year-old boy with high-functioning autism and epilepsy, whereas c.1766G > C/p.Ser589Thr and c.1784G > C/p.Ser595Thr were identified in patients with ASD and ID.

**Fig. 2:**
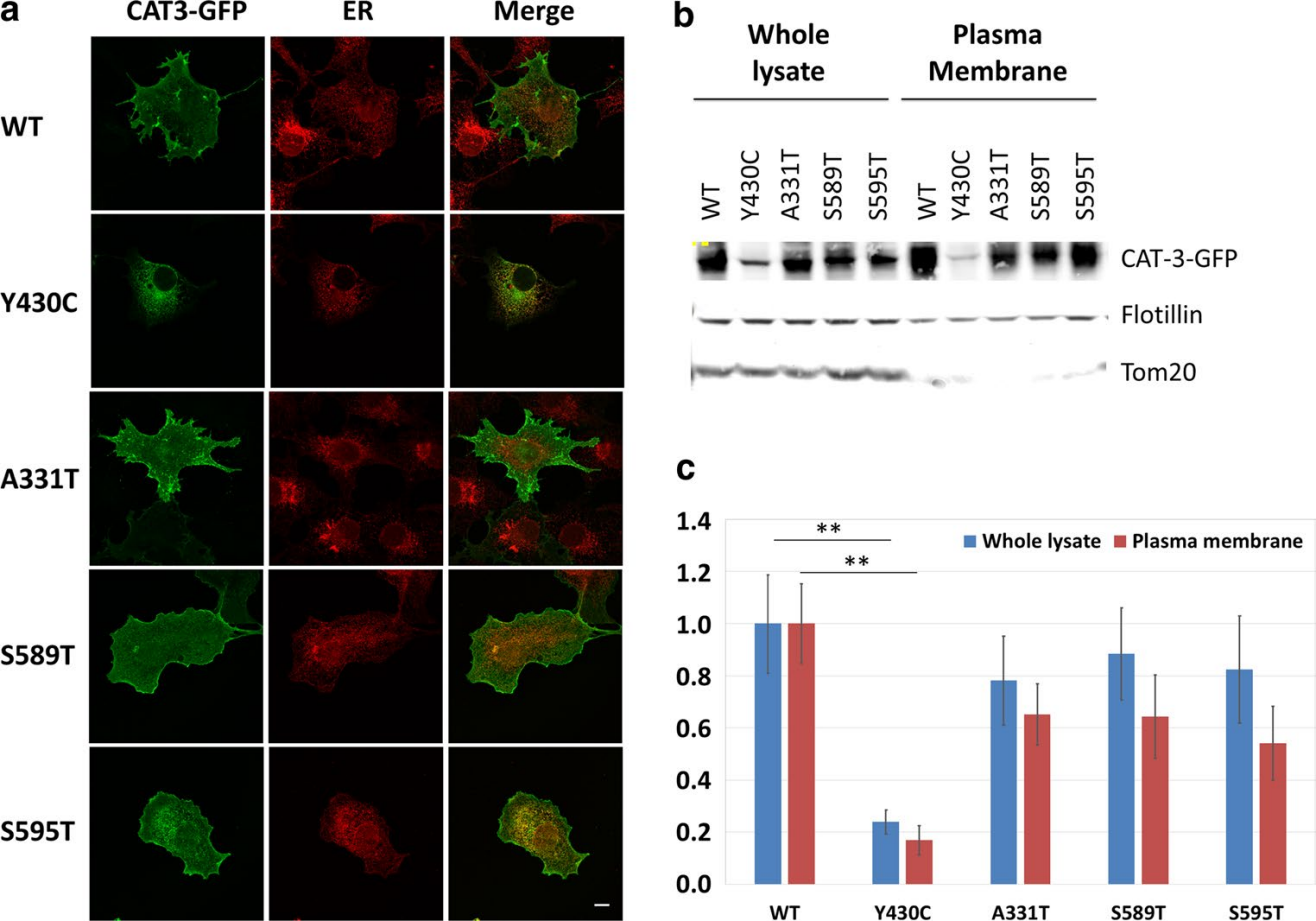
Subcellular localization and expression of CAT-3 mutants at the plasma membrane in mammalian cells. **a** Subcellular localization of wild-type and mutant (p.Tyr430Cys, p.Ala331Thr, p.Ser589Thr, p.Ser595Thr) CAT-3 proteins and colocalization with endoplasmic reticulum (ER, marked using anti-calreticulin) observed by confocal microscopy. *Scale bar* 20 µm. **b** Representative western blot of WT and mutant CAT-3 protein expression in whole lysates and plasma membranes. Flotillin and Tom20 stainings were used to control membrane protein enrichment and normalize protein load, respectively. **c** Quantification of WT and mutant CAT-3 proteins present in whole lysates and plasma membranes. The values, obtained from at least three different experiments, were compared with the Mann–Whitney test; *p < 0.05

To investigate the functional consequences of the identified *SLC7A3* missense variants, we analyzed the cellular distribution and transport activities of the four mutant CAT-3 transporters. We first compared the subcellular localization of transiently expressed WT and mutant CAT-3 proteins fused to GFP in mammalian Cos7 cells. Distribution of p.Tyr430Cys-CAT-3 appeared to be restricted to the endoplasmic reticulum (ER), whereas WT and other types of mutant CAT-3 were mostly present at the plasma membrane (Fig. 2a). By labeling plasma membrane proteins with biotin on the extracellular face of intact cells, we confirmed that the amount of p.Tyr430Cys-CAT-3 at the plasma membrane, as well as the overall amount of protein, decreased strongly in comparison to WT CAT-3 (Fig. 2b, c). These results suggest that p.Tyr430Cys-CAT-3 is unstable or trapped in the ER where it is degraded.

**Fig. 3:**
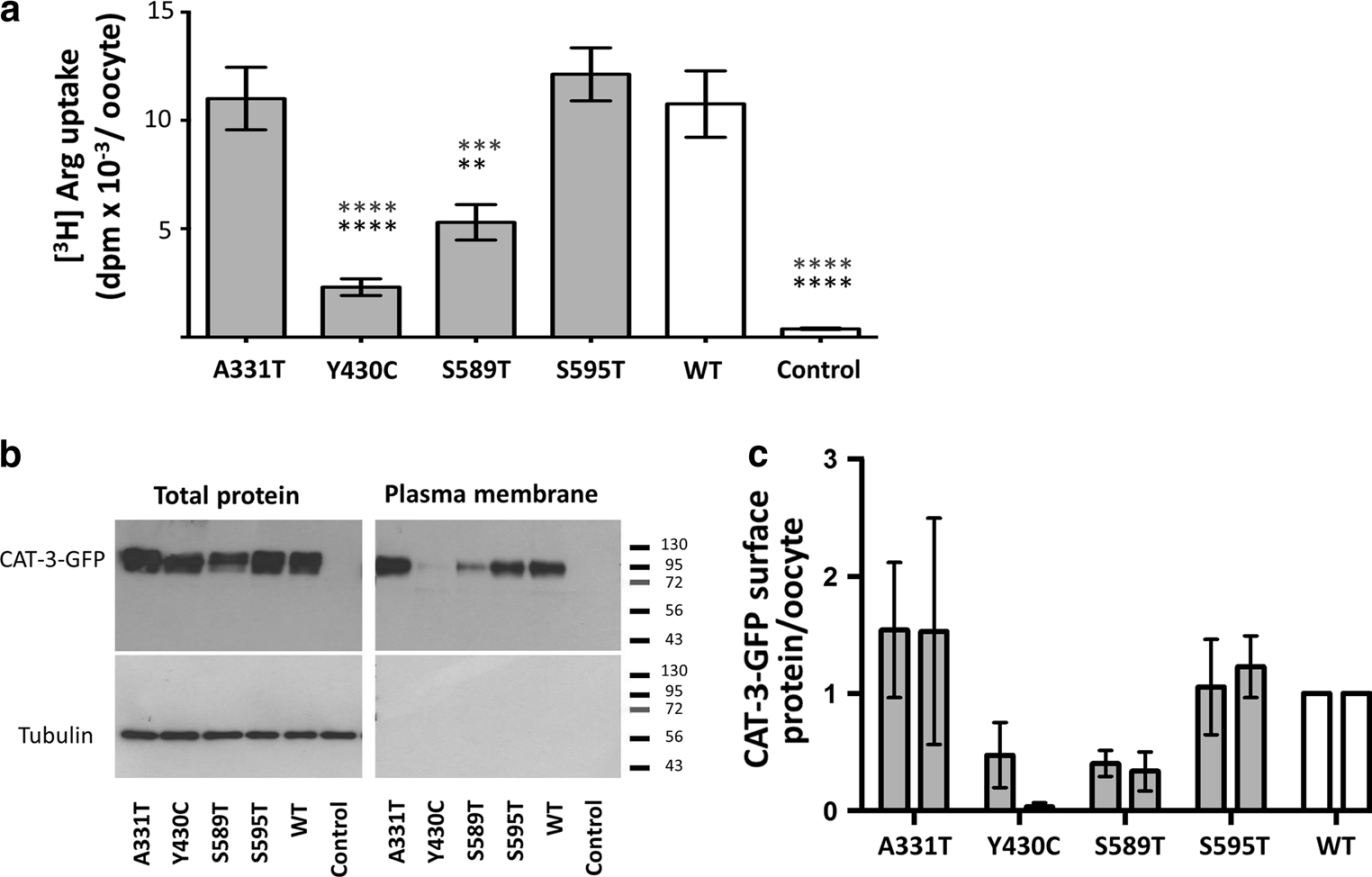
Analysis of WT and mutant CAT-3 transport activity, expression and localization in *Xenopus laevis* oocytes. **a** Transport of 100 µM [^3^H]L-arginine for 15 min into oocytes. **b** Representative Western Blot of WT and mutant CAT-3-EGFP proteins in whole cell lysates and at the plasma membrane. **c** Quantification of three independent experiments as shown in **b**, *left* *columns* CAT-3 in total cell lysate, *right columns* in plasma membrane protein fraction

Further studies in *Xenopus laevis* oocytes showed that transport activities were reduced in oocytes expressing p.Tyr430Cys and p.Ser589Thr compared to oocytes expressing WT CAT-3 (Fig. 3a). Overall, expression of p.Tyr430Cys and p.Ser589Thr proteins was also reduced. The reduction was more pronounced in the plasma membrane fraction, especially for p.Tyr430Cys (Fig. 3b, c). Altogether, these findings confirmed that two of the four *SLC7A3* variants identified had deleterious effects on CAT-3 protein function.

The p.Ala331Thr variant did not alter the cellular distribution of the overexpressed transporter or its transport activity in Xenopus laevis oocytes; we then hypothesized that it may have an effect on CAT-3 that was not detected by the tests we performed. We then assayed arginine, ornithine, and lysine in the CSF of one of the affected brothers with p.Ala331Thr (individual 505-02) sampled in a diagnostic context. Ornithine was slightly decreased in his CSF (5 µmol/L; normal range 7–11 µmol/L), suggesting that a misbalance of cationic amino acids possibly results from CAT-3 dysfunction.

CAT-3 has been reported to be specifically expressed in the brain during embryonic development, suggesting that *SLC7A3* plays a role for in brain development, but this finding is controversial (Hosokawa et al. 1997, 1999; Ito and Groudine 1997; Closs 2002; Vekony et al. 2001; Jager et al. 2013). We therefore developed a quantitative RT-PCR assay to monitor the expression of CAT-3 in the developing mouse brain. *SLC7A3* expression increased from embryonic day 12 (E12) to post-natal day 7 (P7) and declined thereafter; gene expression was strongest in the diencephalon (Fig. S2). In contrast, *SLC7A3* was expressed at very low levels in other tissues including heart and liver. These results are consistent with the expression pattern of *SLC7A3* in the Human Brain Transcriptome Database, which also showed that expression of CAT-3 was higher in the developing human brain.

## Discussion

In this study, we identified missense variants in *SLC7A3,* a gene encoding a CAT specifically expressed in the developing brain, in four male subjects with ASD. We showed evidence that two of the identified variants lead to a severe or moderate loss of function of the CAT-3 transporter. This is the first study showing that hypomorphic *SLC7A3* variants exist in the human male population.

*SLC7A3* is highly intolerant to variation in humans, as shown by the absence of variants introducing premature termination codons in ~61.000 subjects of the ExAc database (http://exac.broadinstitute.org/gene/ENSG00000165349) and the absence of deletion encompassing *SLC7A3* in the DGV database. The absence of variants invalidating *SLC7A3* in thousands of control individuals strongly supports the assumption that complete loss of function of CAT-3 is lethal or pathogenic in humans. Constitutive CAT-3 deficiency was previously hypothesized to be lethal at an early embryonic stage in mammals (Closs et al. 2006). Indeed, CAT-1-deficient mice die rapidly after birth (Perkins et al. 1997), and the spared prenatal development of CAT-1-deficient mice is thought to result from the high levels of CAT-3 expressed in embryonic tissues (Ito and Groudine 1997; Nicholson et al. 1998). In contrast, several deletions encompassing *SLC7A3* among other genes have been reported in affected females in Decipher. A single male patient with a deletion of ~93 Mb encompassing *SLC7A3* (Decipher ID: 284367) is present in Decipher; interestingly, this patient has a syndromic form of autism and developmental delay with additional dysmorphic and neurologic features. In this study, three missense variants were identified out of 148 males with ASD. This proportion (2 %) is higher to the frequency of *SLC7A3* variants observed in the ESP male population (8 ‰, 20/2443) or in male individuals of the IPDGC study (3 ‰, 1/338) (Table S5), although the differences are not significant (*p* = 0.11) due to the small number of variant carriers in each population. However, the probability to observe 3 or more variants among 148 individuals by chance based on the frequency of *SLC7A3* variants in control populations is very low (*p* = 0.05), supporting an excess of *SLC7A3* variants in patients with ASD. The contribution of variants on chromosome X has been well demonstrated in ID but remains unclear in ASD in spite of an excess of affected males in both disorders. Indeed, the most recent studies on ASD genetic factors using whole exome or genome sequencing have focused on de novo mutations and have neglected the role of variants on chromosome X (Murdoch and State 2013; Krumm et al. 2014; O’Roak et al. 2011, 2012; Sanders et al. 2012; Neale et al. 2012; Iossifov et al. 2012, 2014).

**Fig. 4:**
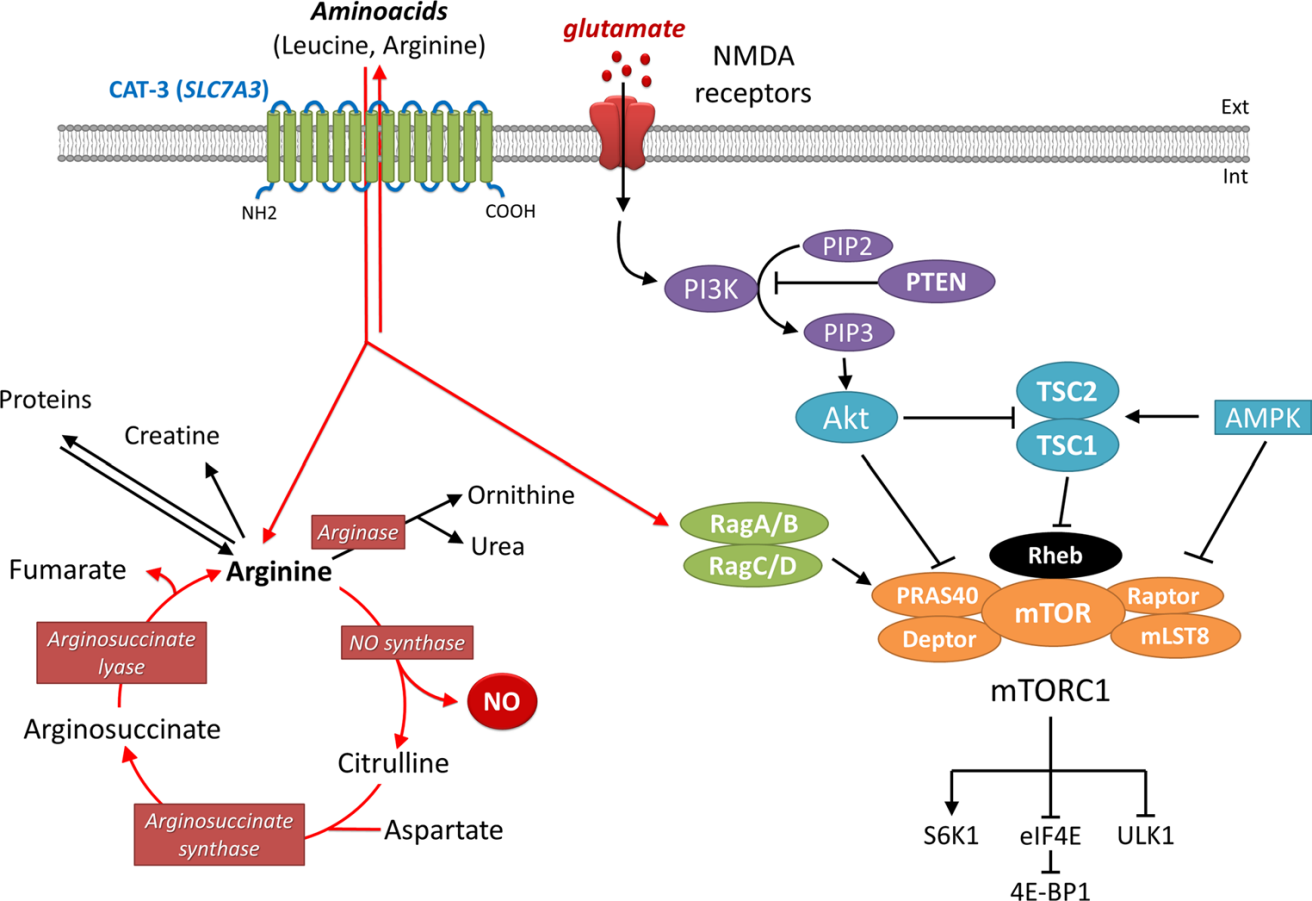
Schematic diagram showing the possible consequences of *SLC7A3* dysfunction on the mTOR and NO pathways. Reduced availability of intracellular cationic amino acids, including arginine, could decrease NO synthesis and alter NO-mediated signaling (*on the left*) or negatively affect the mTOR signaling pathway in neurons (*on the right*)

Cationic amino acid supplies in cells, and therefore CAT-mediated transport, are critical for arginine-, lysine- and ornithine-dependent metabolic reactions. In particular, arginine is the precursor for the synthesis of nitric oxide (NO), creatine and urea, and ornithine is the starting point for polyamine synthesis. In this context, at least two different consequences of *SLC7A3* dysfunction can be hypothesized. On the one hand, since NO is an important cell–cell signaling molecule in the central nervous system (Braissant et al. 1999), reduced availability of arginine in the brain could alter NO synthesis and signaling. In favor of this hypothesis, mice deficient in *Nos1*, which encodes the neuronal NO synthase (nNOS) that converts arginine to NO, display cognitive impairments, aggressivity and hyperactivity as well as additional behavioral abnormalities (Nelson et al. 1995; Weitzdoerfer et al. 2004; Tanda et al. 2009). On the other hand, arginine availability also regulates the mammalian target of rapamycin (mTOR) pathway that controls the survival, differentiation and development of neurons and synaptic plasticity, among other functions (Swiech et al. 2008); reduced CAT-3 activity would therefore be expected to have an impact on the mTOR pathway, which has previously been shown to be impaired in several forms of ASD (Bourgeron 2009; Ehninger and Silva 2011; Veenstra-VanderWeele and Blakely 2012). In particular, CAT-3 variants could modulate the effects of NMDA receptor activation on the mTOR pathway (Fig. 4) (Huang et al. 2007).

Although our results suggest that loss of function of CAT-3 is the main consequence of the identified missense variants, the functional tests performed did not permit to confirm this hypothesis for the p.Ala331Thr, initially identified in Family 505. In spite of the absence of functional effect of this variant on cellular localization and transporter activity, genetic data (absence of this variant from a matched control population, segregation with the disease in the family) support a possible deleterious effect of this variant. In addition, one of the affected brothers had low values of ornithine in his CSF possibly related to a CAT-3 dysfunction. Our hypothesis is therefore that this variant alters an untested function of the CAT-3 transporter. p.Ala331Thr is located in an epitope exposed at the extracellular surface (Fig. 1); it could then alter the interaction of CAT-3 with a putative ligand. Mouse CAT-1 has been shown to be a receptor for retroviruses (Kim et al. 1991). CAT-3 could be a receptor for a cellular signal during development, although this remains to be demonstrated.

The patient with the most deleterious effect on CAT-3 activity (patient 885, p.Tyr430Cys) had typical autism during childhood but his evolution was favorable and he was diagnosed at 10 years old with high-functioning autism. The preferential expression of CAT-3 during embryogenesis suggests that CAT-3 dysfunction has a negative effect during early brain development. This deficit could therefore partially improve or recover with time due to compensatory expression of other CATs, such as CAT-1, as observed for this patient. This patient also had a de novo duplication on chromosome 16p11.2, previously identified by SNP array (Nava et al. 2014b), that possibly contributes to his phenotype. Recurrent reciprocal deletions and duplications involving the 600 Kb 16p11.2 region were repeatedly associated with ASD and schizophrenia, but are characterized by a great phenotypic variability and low penetrance and do not segregate perfectly with ASD in multiplex families (Sanders et al. 2011; Depienne et al. 2009; Weiss et al. 2008; Kumar et al. 2008; Nava et al. 2014b; Levy et al. 2011). The 16p11.2 duplication by itself could then not be considered as the sole cause of ASD in this patient. We hypothesize that the disorder could then result from the association of p.Tyr430Cys in *SLC7A3* and the 16p11.2 duplication, and eventually other variants in the genome. The possibility of oligogenism, suspected in many cases of ASD, has been supported by several recent studies (Schaaf et al. 2011; Barrett et al. 1999; Chilian et al. 2013; Heil and Schaaf 2013; Jiang et al. 2004; Junaid and Pullarkat 2001; Leblond et al. 2012), but identification of the factors interacting together to cause the disorder constitutes a real challenge. Observations on mice suggest that genes functioning in same pathways are more susceptible to display dosage-sensitive genetic interactions (Kidd et al. 1999; Hafezparast et al. 2002). Another possibility is that a burden of rare variants in unconnected genes predisposes to autism in an individual (Veltman and Brunner 2010).

Among the variants shared by the affected sibs in Family 505 was a missense variant (NM_ 000620: p.Arg1369Cys) in *NOS1*. The two variants in *SLC7A3* and *NOS1* could have additive effects, decreasing both the availability of arginine and the conversion of arginine to NO in neurons. Among the variants present in the two brothers possibly contributing to autism was a missense variant in *SCN2A*, also present in their unaffected sister, as well as a missense variant in *CACNA1H* gene, encoding a calcium channel, and missense variant in *FOXP2,* encoding a forkhead-box DNA-binding domain containing transcription factor required for proper development of speech and language, which are both inherited from the healthy mother (Table S3). Variants in *CACNA1H* have previously been identified in patients with ASD, however, they did not segregate with ASD phenotypes, suggesting that they are not causative alone, although they could contribute to the phenotype (Splawski et al. 2006). Mutations in *FOXP2* cause developmental language disorders in humans (Lai et al. 2001). Their contribution to ASD has been extensively been studied (Gauthier et al. 2003; Newbury et al. 2002; Wassink et al. 2002). However, the presence of the missense variant in the healthy mother suggests that this variant is not sufficient to explain the phenotype of the brothers on its own but it could be part of the cause of their language impairment. Further studies are therefore needed to confirm the contribution of *SLC7A3* variants to ASD and apprehend the genetic interactions in individual cases.

## Electronic supplementary material

Supplementary material 1 (DOCX 3942 kb)

Table S3 Rare variants shared by the affected brothers that altered the coding sequence or consensus splice sites (XLSX 48 kb)

## Abbreviations

ASD: Autism spectrum disorders
CAT: Cationic amino acid transporter
CNV: Copy number variants
LOH: Loss of heterozygosity
MAF: Minor allele frequency
mTOR: Mammalian target of rapamycin
NO: Nitric oxide
