# Supplementary material for: Hypomorphic variants of cationic amino acid transporter 3 in males with autism spectrum disorders
Source: Amino Acids. 2015 Jul 28;47(12):2647–58. doi: 10.1007/s00726-015-2057-3 (PMC4633447; doi:10.1007/s00726-015-2057-3)
Supplement: Supplementary file 1 — Supplementary material 1 (DOCX 3942 kb) [file 726_2015_2057_MOESM1_ESM.docx]

**Supporting Information**

**Hypomorphic Variants of SLC7A3 (CAT-3) on Chromosome X in Males with Autism Spectrum Disorders**

Caroline Nava,^1,2,3,4,5^ Johanna Rupp,^6^ Jean-Paul Boissel,^6^ Cyril Mignot^5,7,8,9^ Agnès Rastetter,^1,2,3,4^ Claire Amiet,^10^ Aurélia Jacquette,^5,7,8^ Céline Dupuits,^1,2,3,4^ Delphine Bouteiller,^1,2,3,4^ Boris Keren,^5^  Merle Ruberg,^1,2,3,4^ Anne Faudet,^5^ Diane Doummar,^9^ Anne Philippe,^10^ Didier Périsse,^10,11^ Claudine Laurent,^1,2,3,4,10^ Nicolas Lebrun,^12^ Vincent Guillemot,^13^ Jamel Chelly,^12^ David Cohen,^10,14^ Delphine Héron,^5,7,8,9^ Alexis Brice,^1,2,3,4,5*^ Ellen I. Closs,^6*^ Christel Depienne^1,2,3,4,5*^

**1** Sorbonne Universités, UPMC Univ Paris 06, UMR S 1127, ICM, F-75013, Paris, France ; **2**INSERM, U 1127, F-75013, Paris, France ; **3** CNRS, UMR 7225, F-75013, Paris, France ; **4** Institut du cerveau et de la moelle épinière (ICM), 75013, Paris, France ; **5** AP-HP, Hôpital de la Pitié-Salpêtrière, Département de Génétique et de Cytogénétique, F-75013, Paris, France ; **6**Department of Pharmacology, University Medical Center of the Johannes Gutenberg University, Mainz, Germany ; **7**Centre de Référence "déficiences intellectuelles de causes rares", Paris, France ; **8** Groupe de Recherche Clinique (GRC) "déficience intellectuelle et autisme" UPMC, Paris, France ; **9** AP-HP, Hôpital Trousseau, service de neuropédiatrie, Paris, France ; **10** AP-HP, Hôpital Pitié-Salpêtrière, Service de psychiatrie de l’enfant et de l’adolescent, F-75013, Paris, France ; **11** Centre Diagnostic Autisme de l’Hôpital Pitié-Salpêtrière, F-75013, Paris, France ; **12** Institut Cochin, Inserm U567, UMR 8104, Université René Descartes, Paris 5, France; **13**  Bioinformatics and biostatistics core facility (iCONICS), Institut du cerveau et de la moelle épinière (ICM), Paris, France ; **14** Institut des Systèmes Intelligents et Robotiques, CNRS UMR 7222, UPMC-Paris-6, Paris, France.

**Figure S1** Homozygous regions > 2Mb shared by the affected brothers but not their sister, and the genes present in these regions.


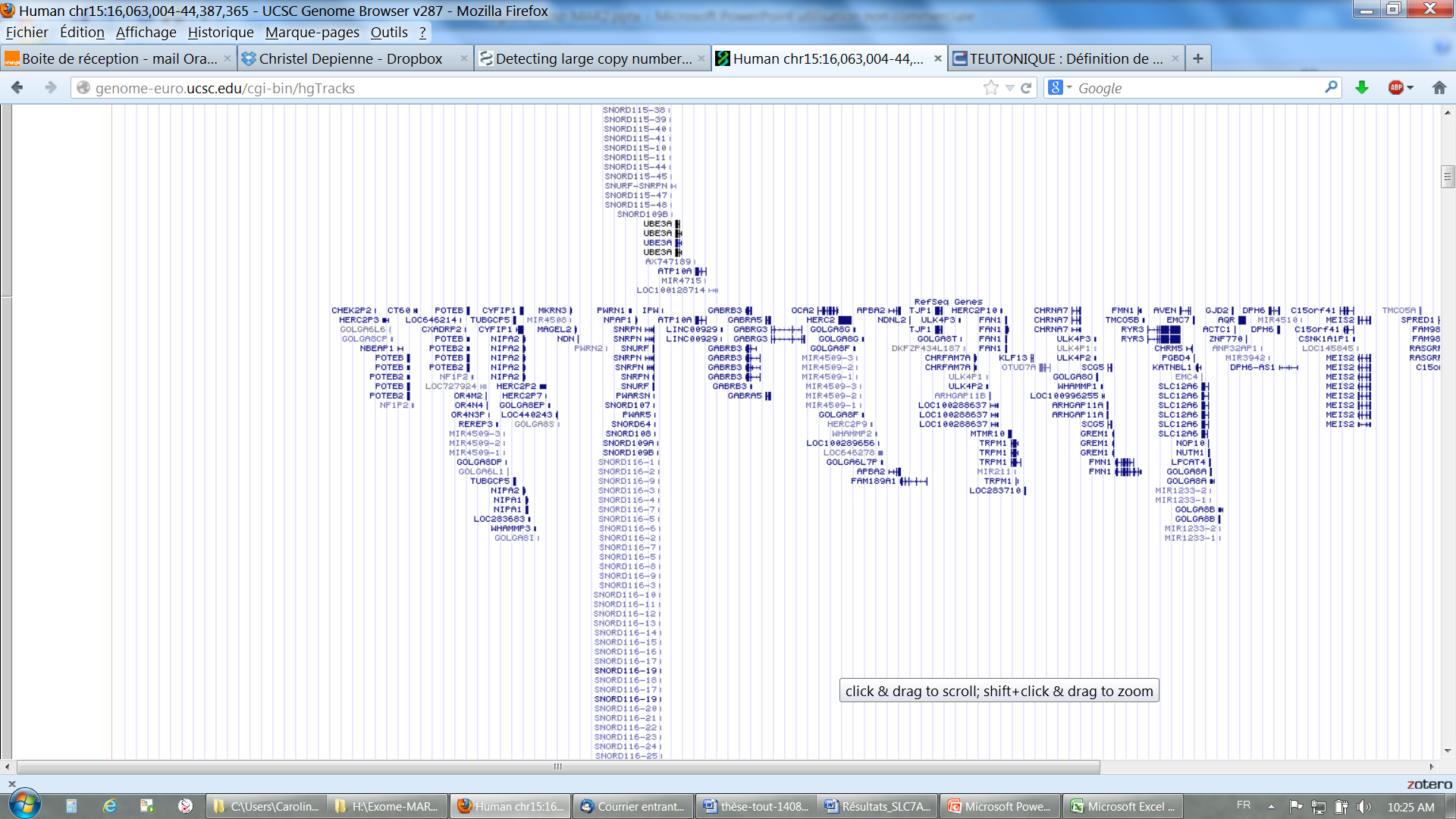

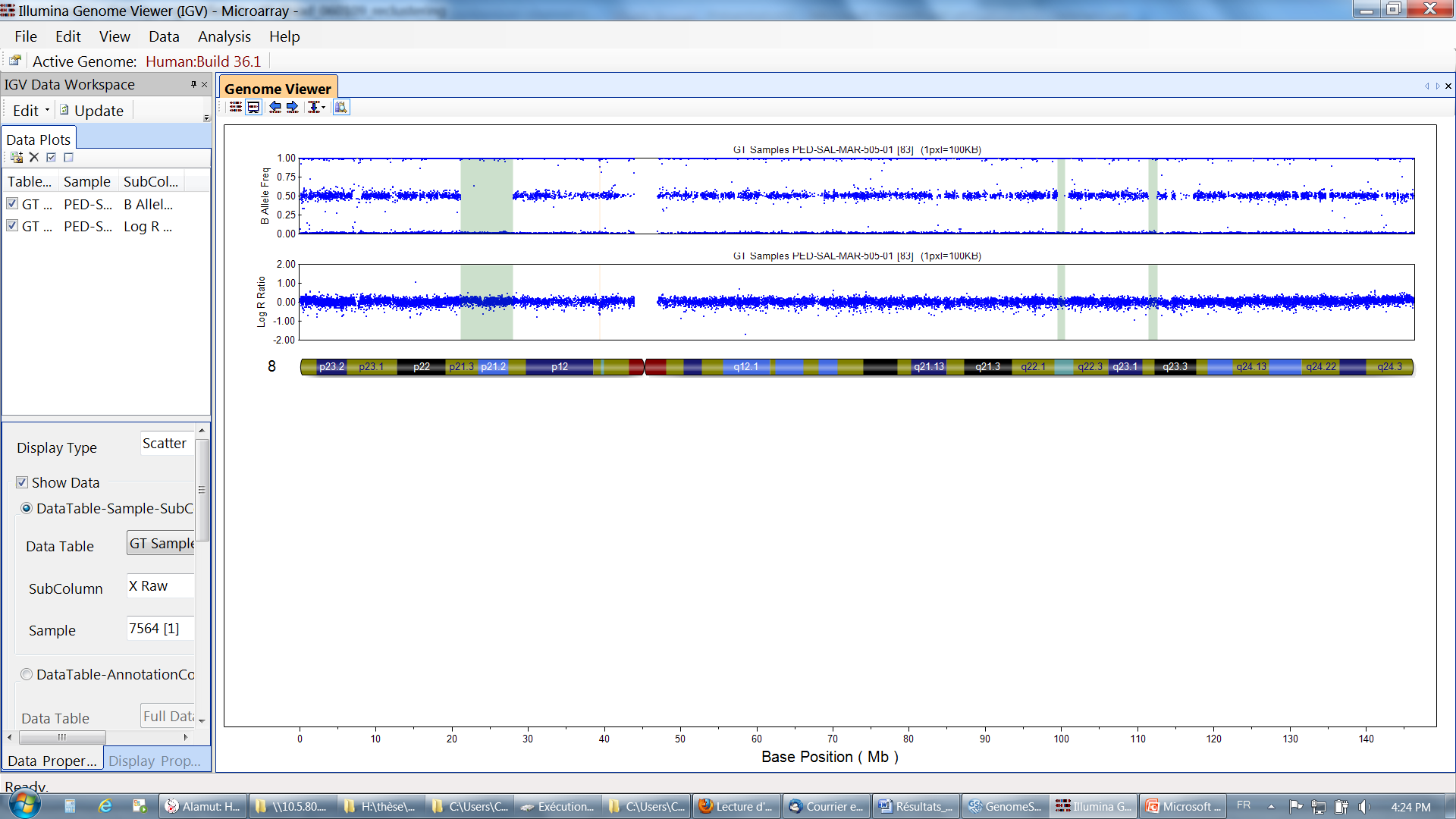

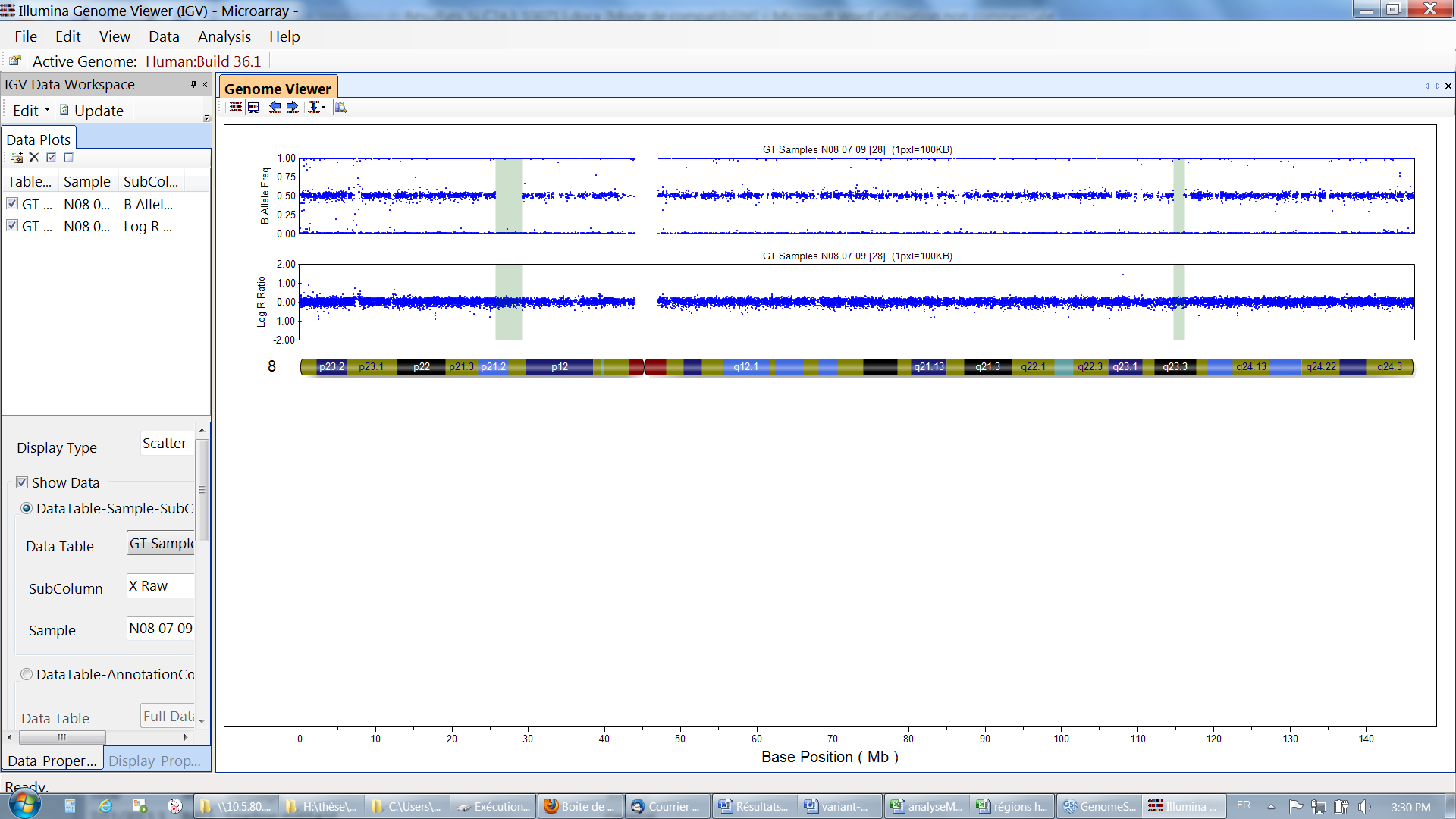

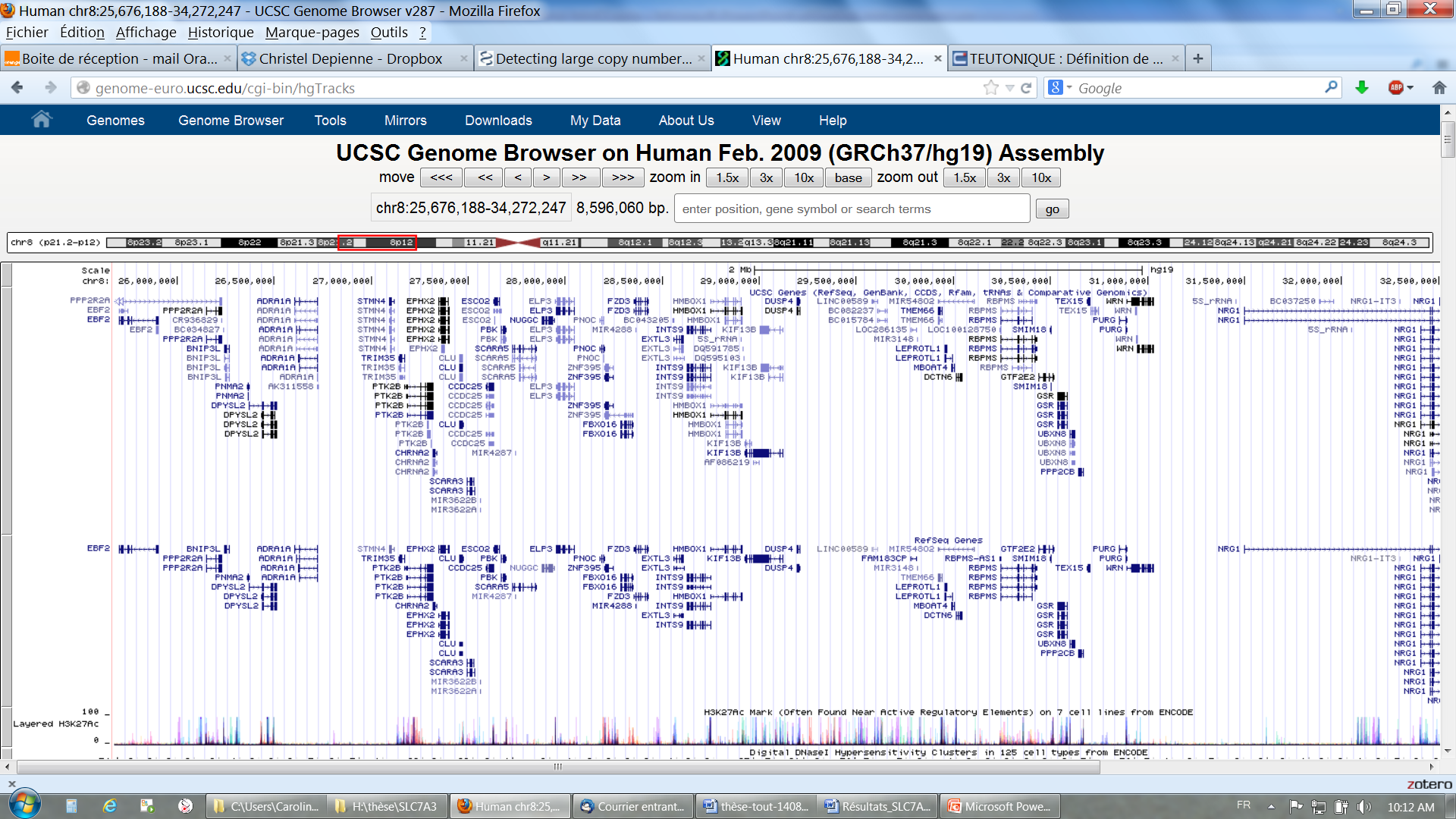

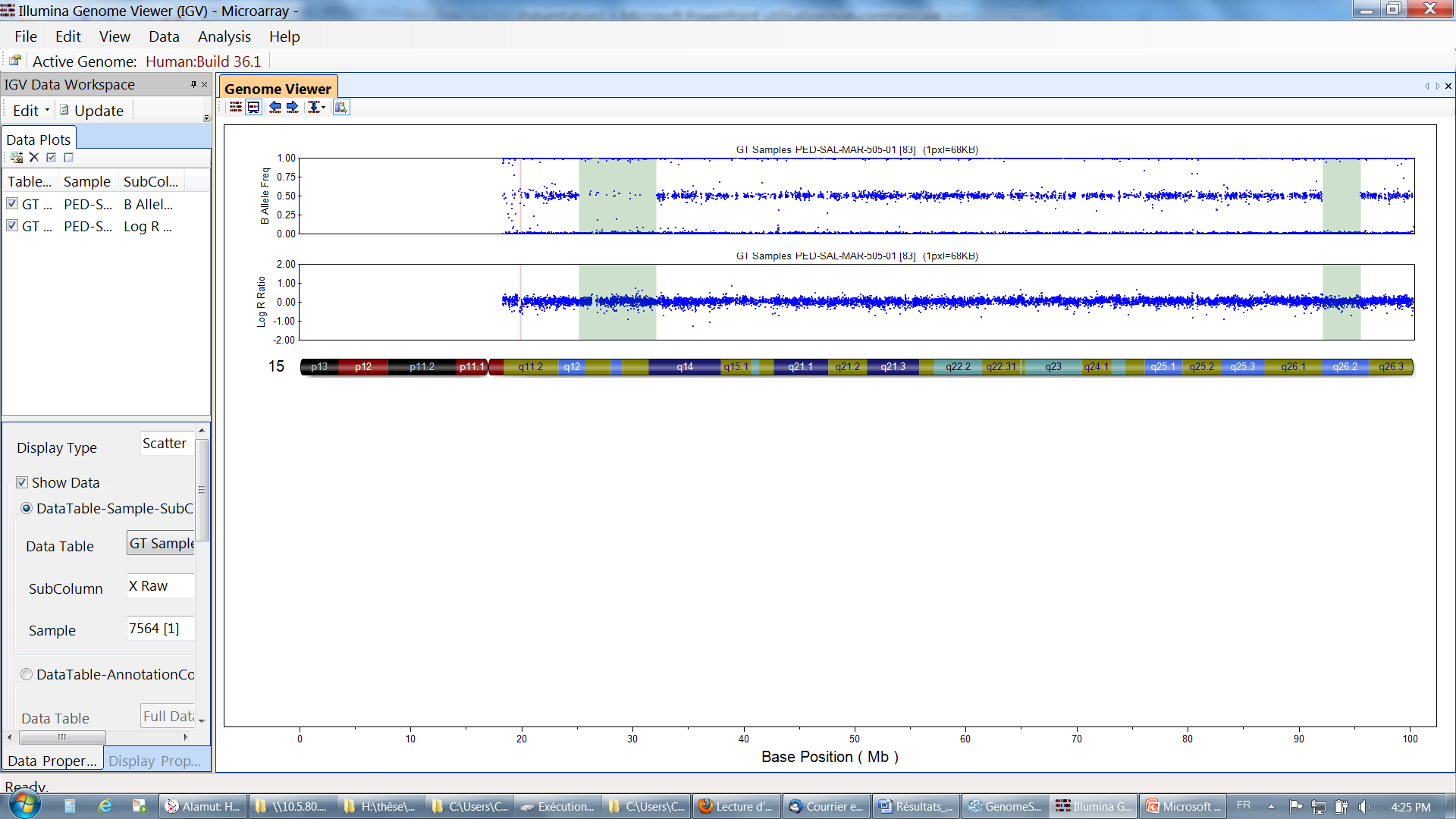

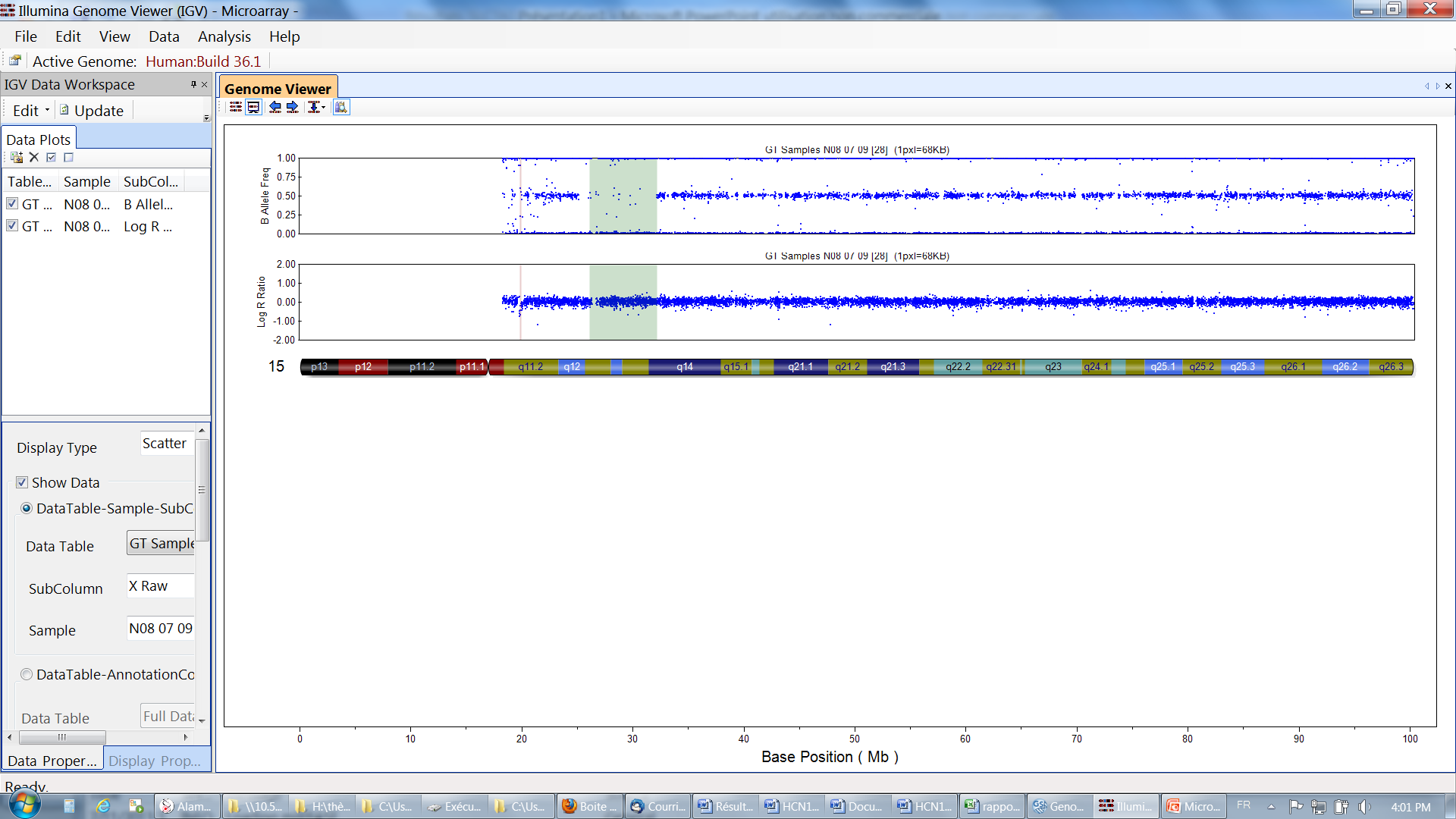


**505-01**

**505-02**

**505-01**

**505-02**

**B Allele Freq**

**Log R Ratio**

**B Allele Freq**

**Log R Ratio**

**Chr 8**

**B Allele Freq**

**Log R Ratio**

**B Allele Freq**

**Log R Ratio**

**Chr 15**

a

b

**Figure S2** Expression of CAT-3 in the developing mouse brain.


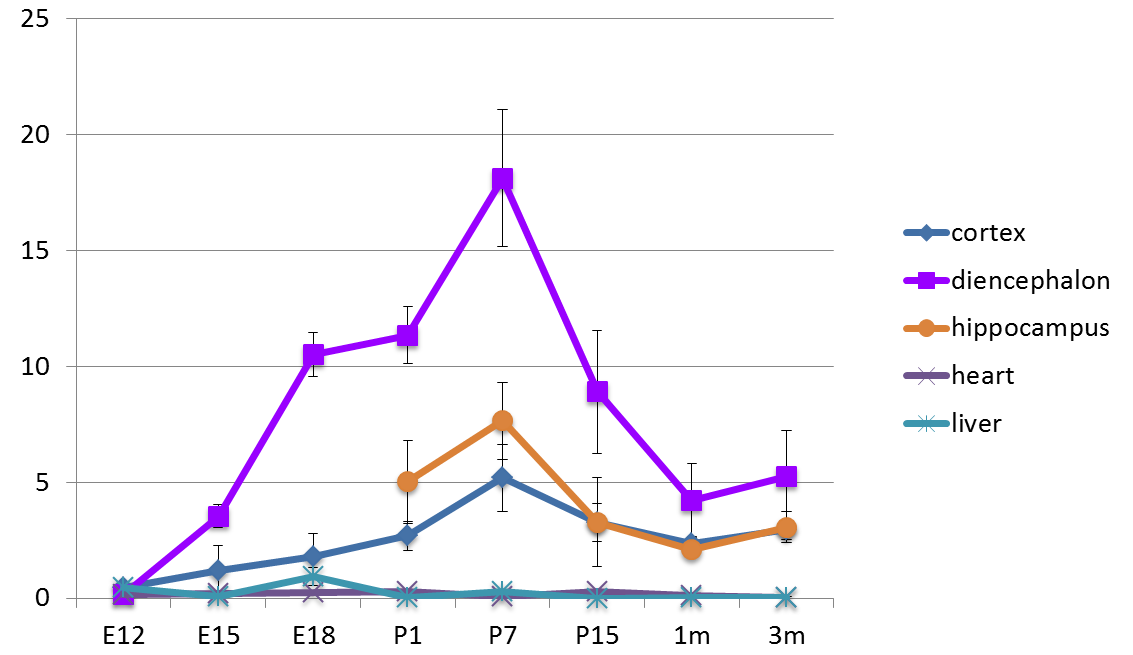


*SLC7A3* expression was assessed on total RNA extracted from the cerebral cortex of wild-type mice at several stages of development (E12, E15, E18, P1, P7, P15, 1 month and 3 months) (36). First-strand synthesis was performed with 120 ng of total RNA using the SuperScript III First-Strand Kit (Invitrogen). Each sample was quantified with the Qiagen QuantiTect primer assay for *SLC7A3*. *PPIA* and *PGK1* were used as control genes. Each sample was run in triplicate on a Lightcycler-1536 apparatus (Roche). Forty-five two-step cycles (15 s at 95°C and 30 s at 60°C) were performed. Quantitative mRNA analysis was performed using qbase Plus software (Biogazelle).

**Table S1** Primer pairs used to amplify the coding exons and intron-exon junctions of *SLC7A3* gene

| **Exon** | **For/rev** | **sequence** |
| --- | --- | --- |
| 2 | Forward | TCATTTCCCAATAGCTCCTTTG |
| 2 | Reverse | ATGAGCAATTTCTCCACACCTT |
| 3 | Forward | AGGTGTGGAGAAATTGCTCATT |
| 3 | Reverse | TCTTCTTCCCCACCATTATCAC |
| 4 | Forward | ATGGGCCTGAGAAAGGCTTA |
| 4 | Reverse | GGAAGAAGGGTTGGAGAAGG |
| 5 | Forward | AGGGGATCTGCTGAAGGATT |
| 5 | Reverse | TCTGGGCTTCTTCTCCTGAA |
| 6 | Reverse | GCAGACTGGGGGTGGTAGAG |
| 6 | Forward | GTCTCCACTTTCCCCTCACA |
| 7 | Reverse | TGTGAGGGGAAAGTGGAGAC |
| 7 | Forward | GAGACCACGGTGGCTATGAT |
| 8 | Reverse | TTTCCTGCCTATTAATTTTA |
| 8 | Forward | TGGAATAATGAAGGCGAAGG |
| 9 | Reverse | GGTATCTCACCCTGCCAAAA |
| 9 | Reverse | GCAGGACAGCTTCAAAGGTC |
| 10 | Forward | GTCGAAGTGTGTGGCATTTG |
| 10 | Reverse | TGCAGTTTGGGTAAATGGAA |
| 11 | Forward | CCCAAACTGCATATTCTTGGA |
| 11 | Reverse | GCCTAGTGGGGAAAGGTAGC |
| 12 | Forward | GGCTTCTTTGGTGTCCTAGC |
| 12 | Reverse | TCAACAAGAAGCACCCACAG |

**Table S2** Homozygous-by-descent regions > 2 Mb shared by the brothers in Family 505 that were heterozygous in their unaffected sister

| Chr | Start (Hg19) | End (Hg19) | Size [bases] |
| --- | --- | --- | --- |
| 8 | 25676188 | 28010554 | 2334366 |
| 15 | 27558300 | 34387365 | 6829065 |

**Table S4** Rare nonsynonymous coding variants on chromosome X, predicted to be deleterious by at least one prediction algorithm and common to the 2 affected brothers of Family 505

| **chr** | **position (Hg19)** | **gene** | **refseq** | **nucleotide change** | **aminoacid change** | **variation type** | **rs number** | **Hapmap Variant Freq (%)** | **EVS (%)** | **EVS genotype** | **1000 Genomes (%)** | **controls** | **SIFT** | **Polyphen2** | **Mutation taster** |
| --- | --- | --- | --- | --- | --- | --- | --- | --- | --- | --- | --- | --- | --- | --- | --- |
| chrX | 48925232 | ***CCDC120*** | NM_001163321 | c.1477G>A | p.Ala493Thr | missense | - | - | 0.0284 | AA=0/AG=2/A=1/ GG=4058/G=2428 | - | 0/171 Eur; 1(male)/133 North African | deleterious | benign | polymorphism |
| chrX | 47426104 | ***ARAF*** | NM_001654 | c.624G>C | p.Gln208His | missense | - | - | - | - | - | 0/130 Eur; 1(male)/111 North African | tolerated | benign | disease causing |
| chrX | 70147700 | SLC7A3 | NM_001048164.2 | c.991G>A | p.Ala331Thr | missense | - | - | - | - | - | 0/190 Eur ; 0/440 North African | tolerated | benign | disease causing |

MAF < 1% (Hapmap, EVS, 1000 genomes and Integragen databases); chr: chromosome; EVS: Exome Variant Server; Eur: European

**Table S5** Summary of *SLC7A3* (NM_001048164.2) variants present in males of the ESP population (Exome variant server) (n=2443) and male controls (n=338) included in the IPDGC study

| Pop | Variant Pos | cDNA Change | Protein Change | Number of males (EA/AA) | rs ID | Impact | SIFT | PolyPhen2 | Mutation Taster |
| --- | --- | --- | --- | --- | --- | --- | --- | --- | --- |
| ESP | X:70145685 | c.1838C>G | p.T613S | 1/0 | rs371854122 | missense | tolerated | benign | polymorphism |
| ESP | X:70145724 | c.1799G>A | p.R600H | 1/0 | rs147665669 | missense | tolerated | benign | polymorphism |
| ESP | X:70145725 | c.1798C>T | p.R600C | 1/0 | rs138954830 | missense | tolerated | damaging | polymorphism |
| ESP | X:70145757 | c.1766G>C | p.S589T | 8/1 | rs149447856 | missense | tolerated | damaging | disease causing |
| CI | X:70147183 | c.1235T>C | p.I412T | 1/0 | rs141687407 | missense | deleterious | damaging | disease causing |
| ESP | X:70147159 | c.1259T>A | p.L420Q | 1/0 | rs200706408 | missense | deleterious | damaging | disease causing |
| ESP | X:70147384 | c.1133C>G | p.T378S | 1/0 | rs151001259 | missense | tolerated | benign | polymorphism |
| ESP | X:70147846 | c.845G>A | p.R282H | 0/1 | rs143349209 | missense | tolerated | benign | polymorphism |
| ESP | X:70148102 | c.713G>C | p.G238A | 0/1 | rs375791608 | missense | tolerated | damaging | polymorphism |
| ESP | X:70148438 | c.575T>C | p.V192A | 0/1 | rs372876920 | missense | tolerated | benign | disease causing |
| ESP | X:70148456 | c.557C>T | p.S186L | 1/0 | rs201201835 | missense | tolerated | damaging | disease causing |
| ESP | X:70148709 | c.514G>T | p.V172L | 0/1 | rs368607994 | missense | tolerated | benign | disease causing |
| ESP | X:70148762 | c.461T>C | p.L154P | 1/0 | rs146198410 | missense | tolerated | benign | polymorphism |

A total of 20 hemizygous missense variants were found in the 2443 cases in the ESP population (frequency of hemizygous variations: 20/2443=0.0082). Only one hemizygous missense variant (I412T) was found in one of the 338 male controls in the IPDGC study. No variants that introduced premature termination codons were found in the hemizygous state in either population. This list does not include the frequent c.1522C>G/p.Leu508Val polymorphism with a MAF is 26.54%.

**Supplementary clinical reports**

**Family 505**

Family 505 comprised 3 children, including two males affected with ID and ASD and their healthy sister, born of a first consanguineous marriage, and three additional children, including a healthy girl and a healthy boy and a young male with learning difficulties, born of two other marriages (Figure 1A).

The proband (01) was born after a normal pregnancy. A materno-foetal infection was reported during the neonatal period. He walked at 12 months, but language was delayed (first words around 6 years). When he was evaluated at 12 years of age, he was able to make simple sentences and write his name. At age 17 years, he was able to make sentences, read and use public transportation. He had autistic spectrum disorder associated with agitation, obsessive-compulsive behaviours and phobias. He also had scoliosis, asthma and sleeping disorders. On clinical examination, his height was +3.5 SD; he had brachycephalia and 2 *cafe-au-lait* spots. The results of the Vineland Adaptive Behaviour Scale at age 17 years gave an age equivalent of 9 years 10 months for communication, 8 years 2 months for socialization, and >18 years 11 months for daily living.

His affected brother (02) made few foetal movements during pregnancy. In the neonatal period, a materno-foetal infection was also reported. He walked at 9 months; behavioural disturbances, including isolation, agitation, aggressiveness, stereotypies and hypersensitivity to noise, were reported from the age of two years. At age 13 years, he had more severe intellectual disability than his brother and had not acquired language. He had sleeping disorders and was treated with Risperidone for behavioural disturbances. The clinical examination was otherwise normal. The results of the Vineland adaptive Behaviour Scale at age 13 years gave an age equivalent of 1 year 5 months for communication, 1 year 6 months for socialization and 4 years 10 months for daily living.

Their younger half-brother (04) had a delay in language development (first words around age 3 years). At age 4 years, he was able to make simple sentences; he attended an ordinary school but had learning difficulties. The results of the Vineland adaptive behaviour scale at age 4.5 years gave an age equivalent of 3 years 3 months for communication, 4 years 2 months for socialization and 4 years 3 months for daily living.

The development of the other three children was unremarkable, language was normal and there were no autistic features.

**Family 885**

The proband (03) was a 10-year-old boy born of unrelated, Caucasian parents. The pregnancy and neonatal periods were unremarkable. He walked at 12 months, but language was delayed: he pronounced his first words at age 3 years and started making sentences at age 7 years. Between 3 and 6 years, he had behavioural disturbances including agitation, stereotypies, echolalia and rituals. He hummed repeatedly and was afraid of noises. Autism was diagnosed at 5 years of age. At 9 years of age, he started having temporal seizures. His evolution was favourable, however: at age 10 years, he attended an ordinary school, had normal cognitive abilities and his social relationships had improved. Autism without intellectual disability was diagnosed. He had no dysmorphy, and his brain MRI was normal. Analysis with SNP-arrays found a *de novo* 16p11.2 duplication.

**Family 388**

The proband (individual 02) was a 14 year-old boy of North African origin. He was the first of two children, and his parents were unrelated. His sister had a moderate intellectual disability without behavioural disturbances. The intellectual disability of the proband, who did not acquire language, was more severe than his sister's and was associated with autistic spectrum disorder, agitation and heteroagressivity.

**Family 962**

The proband (individual 03) was a 7 year-old boy born of unrelated, Chinese parents. He has a healthy sister. His mother received Carbimazole for hypothyroidism during pregnancy. His neonatal period was unremarkable; he walked at 11 months. He was diagnosed with autistic spectrum disorder at age 2 years. At 7 years, language had not been acquired, and his height was > +2 SD.
